# Cortical Distance but Not Physical Distance Modulates Attentional Rhythms

**DOI:** 10.3389/fpsyg.2020.541085

**Published:** 2020-11-19

**Authors:** Airui Chen, Guangyao Zu, Bo Dong, Ming Zhang

**Affiliations:** ^1^Department of Psychology, Suzhou University of Science and Technology, Suzhou, China; ^2^Department of Psychology, Soochow University, Suzhou, China; ^3^Cognitive Neuroscience Lab, Graduate School of Interdisciplinary Science and Engineering in Health Systems, Okayama University, Tsushima-Naka Campus, Okayama, Japan

**Keywords:** attentional rhythms, cortical distance, visual cortical areas, high-temporal-resolution, cue-target paradigm

## Abstract

It has been well documented that the spotlight of attention is intrinsically rhythmic and oscillates by discretely sampling either single or multiple objects. However, the neural site of attentional rhythms remains poorly understood. Considering the topography of visual cortical areas, we modulated the cortical distances of two gratings while fixing the corresponding retinal distance by setting the gratings on different sides (cortically far, Experiment 1) or on the same side (cortically near, Experiment 2) of the vertical median, to investigate the interhemispheric divide effect in attentional rhythms. The cue-target stimulus onset asynchrony (SOA) varied from 0.1 s to 1.08 s in 20-ms increments, allowing fluctuations below 50 Hz to be examined. The results showed that when the two stimuli were on opposite sides of the vertical meridian, attentional rhythms were observed at theta and alpha frequencies, consistent with the results reported in previous studies. However, when the two stimuli were located on the same side of the vertical meridian, attentional rhythms were not observed. This study indicates that attentional rhythms are modulated by cortical distance but not by physical distance.

## Introduction

Visual attention samples information discretely rather than continuously; thus, researchers have observed fluctuations in both behavioral performance and neural activity (VanRullen, 2016, 2018). The behavioral correlates of attentional rhythms (including accuracy, reaction time, reverse frequency of continuous wagon wheel illusions, and saccadic eye movements) have been extensively investigated (VanRullen, 2007; VanRullen et al., 2007, 2014; Busch and VanRullen, 2010; Landau and Fries, 2012; Fiebelkorn et al., 2013; Song et al., 2014; Drewes et al., 2015; Dugué et al., 2015, 2017; Chen et al., 2017a,b, 2018; Benedetto and Morrone, 2019; Zhang et al., 2019). Although studies have suggested that some frequencies (e.g., 7-Hz frequencies and 4-Hz frequencies in the calcarine sulcus, lingual gyrus, and precuneus gyrus and the V1/V2 visual cortical regions) are correlated with visual attentional rhythms (Busch and VanRullen, 2010; Landau et al., 2015; Dugué et al., 2016), the cortical characteristics that govern these oscillations remain poorly understood.

In the current study, we investigate whether cortical distance influences attentional rhythms in the absence of artificial intervention by taking advantage of the dissociation between retinal distance and cortical distance. As is well known, neurons corresponding to the left (right) visual field transmit visual information to the right (left) hemisphere of the cerebral cortex according to the retinotopic mapping relationship between the retina and visual cortex (Rajimehr and Tootell, 2009; Abrams et al., 2012). This relationship means that the visual cortex in one side of the hemisphere represents the contralateral visual hemifield. When the two stimuli are located on opposite sides (e.g., the left and right visual hemifields) of the vertical meridian (VM), they are projected to the left and right primary and other early visual cortices, and they are cortically far from each other because their interactions must pass through the corpus callosum. However, stimuli located on the same side (e.g., the left visual hemifield) of the VM are represented in the ipsilateral visual cortex area (the early visual cortices on the right side of the brain) and are cortically near each other (Sereno et al., 1995; Deyoe et al., 1996). Thus, two visual stimuli are cortically near if they are located on the same side of the visual field and cortically far if they are distributed such that one is on each side of the visual field, even if they are located at the same physical (retinal) distance under these two conditions.

Two experiments were designed to investigate whether this interhemispheric divide effect exists for attentional rhythms. The cortical distance between two stimuli was manipulated by presenting the stimuli either on opposite sides (Experiment 1) or on the same side (Experiment 2) of the VM while maintaining an identical distance of each stimulus from the retina. All gratings were located in the lower peripheral visual field rather than in the middle of the vertical field; thus, the horizontal meridian was not involved. To examine attentional rhythms, we adopted the high-temporal-resolution cue-target paradigm, which has been used in previous studies to investigate the temporal characteristics of visual attention (Landau and Fries, 2012; Song et al., 2014; Chen et al., 2017a). In the task, the cue and target sequentially occurred on either grating with 50% cue validity, and 50 stimulus onset asynchrony (SOA) levels were set up to measure fluctuations in behavioral performance. If attentional rhythms are modulated by an interhemispheric divide effect, the results of Experiments 1 and 2 should show different patterns. Fourteen subjects were randomly recruited for each of the two experiments, and three of the subjects participated in both experiments; thus, a total of 25 subjects participated in this study.

## Experiment 1: Attentional Rhythms Under Far Cortical Distance Conditions

In Experiment 1, we examined attentional rhythms when gratings were represented across the VM and designed a single-factor, two-level (spatial validity: cued vs. uncued) experiment. To examine attentional rhythms, 50 SOA levels (ranging from 100 to 1080 ms in 20-ms increments with a sampling frequency of 50 Hz) were used. In this experiment, the temporal characteristics of behavioral fluctuations were calculated by measuring the accuracy at each SOA level.

### Methods

#### Participants

Fourteen college students (2 male and 12 female participants, 19–29 years of age and all right-handed) were recruited for the experiment and provided written consent. All subjects had normal or corrected visual acuity and no color blindness or color weakness. The participants were paid after the experiment. This study was conducted in accordance with the Declaration of Helsinki and approved by the ethical committee of Soochow University.

#### Apparatus and Stimuli

The experimental program was coded using MATLAB and Psychophysics Toolbox-3 (Pelli and Zhang, 1991; Brainard, 1997; Pelli, 1997) and performed using a Dell OptiPlex 755 computer connected to a 22-inch ViewSonic P225f CRT display with a resolution of 1024 × 768 and a refresh rate of 100 Hz. Responses were recorded using a keypad.

**FIGURE 1:**
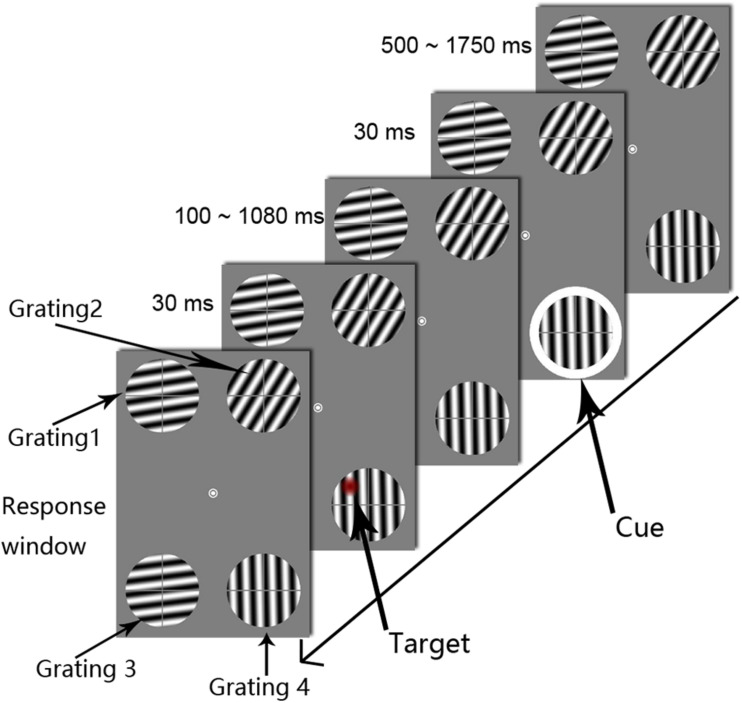
The procedures used in Experiment 1. Each trial display showed four drifting gratings, the orientations of which were randomized across trials. The white circle was the cue stimulus. The target appeared at one of the 50 temporal intervals, in steps of 20 ms, from 0.1 to 1.08 s after cue onset (SOA). The subjects were asked to determine the target’s location. To clearly illustrate the procedure, the target is marked as a red circle.

The experimental method and parameters were based on those reported by Landau and Fries (2012) and modified according to the objectives of this study. Specifically, all stimuli were presented on a gray background at a luminance of 3.88 cd/m^2^ (Figure 1). The fixation point was a white annulus (0.5° in diameter) in the center of the viewing screen. Four gratings were displayed peripheral to the central fixation point. All of the gratings were 3° in diameter and 6° in distance from the central fixation point (eccentricity). The spatial frequency and the contrast of the gratings were 1.4 c/° and 100%, respectively. One grating was located in each of the following areas: the upper left, upper right, lower left, and lower right portion of the screen. The central fixation point (0°, 0°) was set as the center of the reference coordinate system, and the *x*-axis represented the horizontal direction of the screen, while the *y*-axis represented the vertical direction. Grating 1 was in the upper left area of the screen, with center coordinates of (−2.8°, 5.3°); grating 2 was in the upper right area of the screen, with center coordinates of (2.8°, 5.3°); grating 3 was in the lower left area of the screen, with center coordinates of (−2.8°, −5.3°); and grating 4 was in the lower right area of the screen, with center coordinates of (2.8°, −5.3°). The distance between the centers of gratings 3 and 4 was 5.6° visual angle. The target and cue stimuli appeared only on gratings 3 and 4; gratings 1 and 2 were presented to help avoid active eye movement strategies. In the preliminary experiment, we only presented gratings 3 and 4. However, the participants reported that this presentation attracted their eyes to the lower gratings and caused them to look down at the lower portion of the screen. Therefore, we modified our presentation to include four symmetrical gratings to help the participants avoid eye movement. To prevent visual adaptation to the grating orientation, the four grating directions were randomly chosen from 0° to 360° separately in each trial. To minimize the effect of attention capture of the target stimulus on attentional rhythms, the grating was not maintained in a fixed phase but was instead moved in a specific direction. In each trial, all of the gratings moved at a speed of 0.7 c/s, and the direction of motion was perpendicular to the orientation of the grating. Moreover, each grating was divided equally into an upper left quadrant, an upper right quadrant, a lower left quadrant, and a lower right quadrant by a gray cross consisting of lines 0.22° in thickness. As shown in Figure 1, the target appeared in one of the four quadrants. The cross line was used to discriminate the target’s relative location. The subject was asked to complete a four-alternative forced choice (4AFC) task that involved determining the location of the target stimulus (i.e., on which sector of the grating did the target stimulus appear?). The cue stimulus was a white annulus with an inner diameter of 3° and a ring width of 0.2°. During the experiment, the cue appeared randomly on the outer periphery of grating 3 or 4 with a presentation time of 30 ms. The target stimulus refers to an abrupt decrease in contrast in any given circular area (1° in diameter) on the grating. Similarly, to reduce the dominant effect of the target stimulus, the contrast decrement values in the entire circular region were multiplied by a Gaussian distribution with a standard deviation (SD) of 0.5°. The value of the maximum decrement was determined using an adaptive step method (QUEST). The target stimulus had a presentation time of 30 ms and appeared randomly in one of the four regions within the two lower gratings, and overlap with the cross line was avoided.

#### Procedure and Design

The experiment was conducted in a dark room. The subjects were asked to sit in front of the screen at a distance of 50 cm from the screen with their chins resting on a chin rest. To better detect attentional discreteness, a suitable degree of difficulty was determined for each subject by using the QUEST method and setting the probability of the subject’s detecting the target stimuli to 50% (i.e., the threshold measurement) and then examining the periodic changes in the subject’s detection of visual stimuli under cued and uncued conditions (i.e., oscillation measurement).

Prior to the oscillation measurement phase, the subject was informed that the target stimulus would appear within one of the two gratings in the lower left and lower right regions of the screen and not within either of the two gratings in the upper left and upper right regions of the screen. The subject was also informed that the cue annulus and the target stimulus might be located within the same grating or in two different gratings in the lower part of the screen, with a 50% probability of each. In each trial, the fixation point and the four moving gratings were first presented for 1000–1200 ms, and the cue stimulus then appeared in the outer periphery of a grating in the lower part of the screen for 30 ms. The target stimulus then appeared only on grating 3 or grating 4 for 30 ms; the fixation point and the moving gratings were continuously represented on the screen until the subject entered a response by pressing a key. The cue-target SOA varied from 0.1 s to 1.08 s in 20-ms increments, allowing fluctuations below 50 Hz to be examined. To reduce the difficulty of the key-pressing task, we chose the 1, 2, 4, and 5 keys on the number keypad of the keyboard to correspond to the four parts of the grating (1 for lower left, 2 for lower right, 4 for upper left, and 5 for upper right). It should be noted that the subject was asked to ignore the question of which grating the target stimulus appeared in and was only required to determine the region of the grating in which the target stimulus appeared. If the subject did not respond by pressing a key within 3 s, a prompt in black letters (“lower left, lower right, upper left, or upper right”) was displayed until the subject pressed a key. After the subject pressed a key, feedback was provided by displaying either “correct” or “incorrect” in the center of the screen. Therefore, if the participant completely missed the target, they were also forced to make a judgment. Because the target’s visibility in the experiment itself was set at 50% using the QUEST procedure, this task was quite difficult. Even if the participant focused on the gratings, there was not a high probability that he or she would see the target. Only when the participant correctly reported the location of the target among the four possible positions was the response identified as correct; pressing one of the other adjacent buttons was marked as an incorrect response. During the oscillation measurement phase, each subject completed 32 trials at each SOA level (16 cued trials, 16 uncued trials, and no catch trials). There were 50 SOA levels. Thus, there were 1,600 trials in total. To avoid a fatigue effect, the oscillation measurement phase was divided into eight test sessions (200 trials in each session) that were completed over a period of 2–3 days. Participants could rest after every 67 trials in each session. They were asked to complete at least two sessions and at most four sessions per day.

The contrast decrement values of the target stimulus were measured during the threshold measurement phase. With the sole difference that a cue stimulus was not presented, the stimuli and the procedure used for the threshold measurement were identical to those described for the oscillation measurement phase. The program automatically adjusted the contrast decrement values for the target location based on the subject’s responses measured using the QUEST procedure. The greater the decrement was, the more intense the target stimulus was, and vice versa. If the subject was able to correctly judge the location of the target stimulus, the contrast decrement in the subsequent test was reduced; otherwise, the contrast decrement was increased. This procedure facilitated the determination of the appropriate threshold (i.e., the point at which the subject correctly detected the location of the target stimulus with a probability of 50%) for the subject to detect the target stimulus. There were 60 trials in each QUEST test and three QUEST tests each for gratings 3 and 4. Thus, there were 360 trials in the threshold test. The mean threshold levels of the three QUEST tests for each grating were used to determine the contrast decrement values of the target stimulus for gratings 3 and 4, respectively, during the oscillation measurement phase.

#### Data Analysis

The accuracy data of the subjects were analyzed using MATLAB and the CircStat Toolbox. First, the accuracies at each SOA under cued and uncued conditions were calculated. For example, in Experiment 1, there were 16 trials under the cued condition when the SOA level was 100 ms. If a participant pressed the correct key in the 4AFC task (the choices were the 1, 2, 4, and 5 keys on the number keypad of the keyboard), the trial was marked as a correct trial. We then divided the number of correct trials under this condition by 16. If the participant made the correct response in eight trials, the accuracy at this SOA level was 50%. Second, the respective accuracies under cued and uncued conditions were sorted based on SOA (from 100 to 1080 ms) to obtain the pattern of temporal fluctuations (i.e., behavioral oscillations) in the subject’s detection ability (ACC-SOA signal). Third, to analyze the spectral characteristics of behavioral oscillations, the behavioral oscillations of each subject were determined using spectral analysis. Specifically, after preprocessing with zero pad, detrend, and Hanning tapers, the temporal domain of behavioral oscillations was transformed to a frequency domain through the fast Fourier transform (FFT) for each condition. The entire constructed time course was utilized in the FFT analysis. This analysis allowed the oscillation information within the behavioral oscillations to be detected. In addition to the frequency, we also calculated the phase information for behavioral oscillations under both cued and uncued conditions to further determine the oscillation pattern. The phase information for each subject was extracted at each frequency (0–25 Hz), and the phase values for the cued condition were then subtracted from the phase values for the uncued condition to determine the phase differences between the two conditions. Finally, the cross-subject coherence in the phase difference values was calculated for each subject to clarify the phase relationship between the cued and uncued conditions at each frequency, and the significant levels of the inconsistencies in the phase information at each significant frequency band were tested using the Rayleigh test.

To determine the frequency at which significant oscillations occurred, the following non-parametric statistical method was adopted. First, for each subject, for each condition, the time information in the ACC-SOA signal was randomly shuffled 10,000 times to generate 10,000 surrogate signals. For each surrogate signal, the above-described FFT analysis was performed to obtain the corresponding amplitudes for each of the 10,000 surrogate signals at each frequency, and these data constituted the permutation distribution of the frequency domain information. The permutation distribution and oscillation information of the original behavioral oscillations were analyzed using the permutation test, and the significant levels were determined for the oscillations at each frequency (0–25 Hz). Because the permutation test involved multiple comparisons, the more stringent Bonferroni method was used to correct the results to avoid false-positive results. The denominator dividing the critical *p*-values is 25 (e.g., *p* = 0.05/25 = 0.002).

### Results and Discussion

**FIGURE 2:**
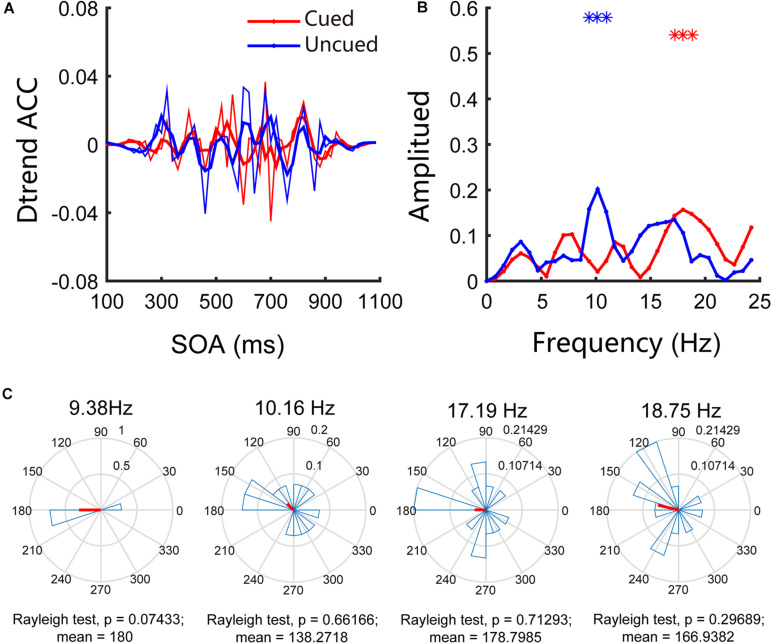
Behavioral oscillations under the far cortical distance condition. **(A)** Under both cued and uncued conditions, accuracy changed with SOA. **(B)** Amplitude of each frequency band under cued and uncued conditions. Red and blue asterisks indicate the significant frequency bands. **(C)** Phase coherence between the cued and uncued conditions. Each subject’s phase difference is plotted on the circle; the average difference is plotted.

Similar to the traditional 4AFC task in psychophysics research, our experiment focused only on accuracy and not on reaction time. In Experiment 1, we examined the attentional rhythms in the far cortical distance condition. The accuracies in the cued and uncued conditions were 58.32% and 55.04%, respectively, and the paired *t*-test results were *t*(13) = 1.44, *p* = 0.17, and Cohen’s *d* = 0.30, indicating that the accuracies in the two conditions are not significantly different. To further investigate the pattern of attentional rhythms in the far cortical distance condition, we performed Fourier analysis and permutation tests; the results indicated an apparent behavioral oscillation pattern (see Figures 2A,B). Further analysis of the behavioral oscillations revealed that significant oscillation bands were observed under both conditions. Specifically, under the cued condition, significant oscillations occurred at 18.75 Hz (*p* < 0.05/25), whereas under the uncued condition, significant oscillations occurred at 9.38 Hz, 10.16 Hz, and 17.19 Hz (*p* < 0.05/25). Figure 2C shows the results of phase analysis. The phase relationships at other frequencies were not significant except for a marginally significant difference around 180° (*p* = 0.07) at 9.38 Hz.

## Experiment 2: Attentional Rhythms Under Near Cortical Distance Conditions

In Experiment 2, we examined the attentional rhythms that occurred when the stimuli were presented on the same side of the VM at the near cortical distance with a retinal distance identical to that in Experiment 1. A single-factor, two-level (spatial validity: cued vs. uncued), within-subjects experimental design was applied. In this experiment, the target and the cue stimulus were ipsilaterally represented in the lower right side of fixation. Previous research has shown that the antiphase pattern in the human right visual field is more evident than that in the left visual field (Landau and Fries, 2012). Therefore, in Experiment 2, we tested attentional rhythms in the right visual field rather than in the left visual field. Specifically, in contrast to the positions of the gratings in Experiment 1, the two gratings, on which the target and cue occurred, were both placed in the lower right visual field in Experiment 2; this would activate the left cortical hemisphere. In the cued condition, the cue and the target appeared on the same grating in the lower right area of the screen. In the uncued condition, the cue and the target appeared on different gratings in the lower right area of the screen. The physical distance between the centers of the lower left and lower right gratings was 5.6°, the same as the distance in Experiment 1, but the stimuli were presented to neurons with near cortical distances in visual areas.

### Methods

**FIGURE 3:**
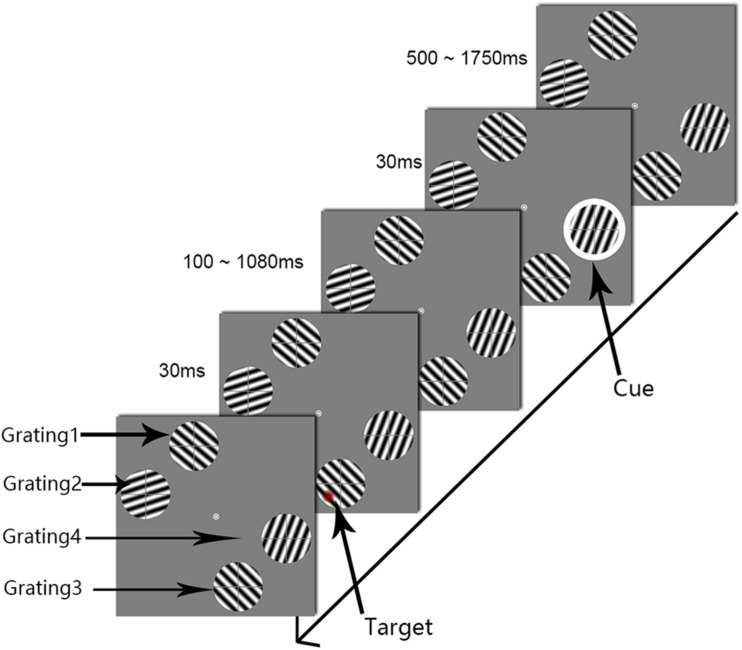
The procedure used in Experiment 2. The white circle was the cue stimulus. The target appeared at one of the 50 temporal intervals, in steps of 20 ms from 0.1 to 1.08 s after cue onset (SOA). To clearly illustrate the procedure, the target is marked as a red circle.

Experiment 2 differed from Experiment 1 in the locations of the four gratings (see Figure 3). Taking the central fixation point (0°, 0°) as the center of the reference coordinate system, the horizontal direction of the screen was the *x*-axis, the vertical direction was the *y*-axis. The two gratings (gratings 1 and 2) were in the upper left area of the screen. Grating 1 had center coordinates of (*−*1.8°, 5.7°), and grating 2 had center coordinates of (*−*5.7°, 1.8°). The other gratings (gratings 3 and 4) were located in the lower right area of the screen: grating 3 had center coordinates of (1.8°, *−*5.7°), and grating 4 had center coordinates of (5.7°, *−*1.8°). The distance between the centers of gratings 3 and 4 was 5.6°, the same retinal distance as was used in Experiment 1; however, in this experiment, the cue and the target were projected to cortical areas with a near cortical distance. The other stimulation parameters were the same as those described in Experiment 1.

### Results and Discussion

**FIGURE 4:**
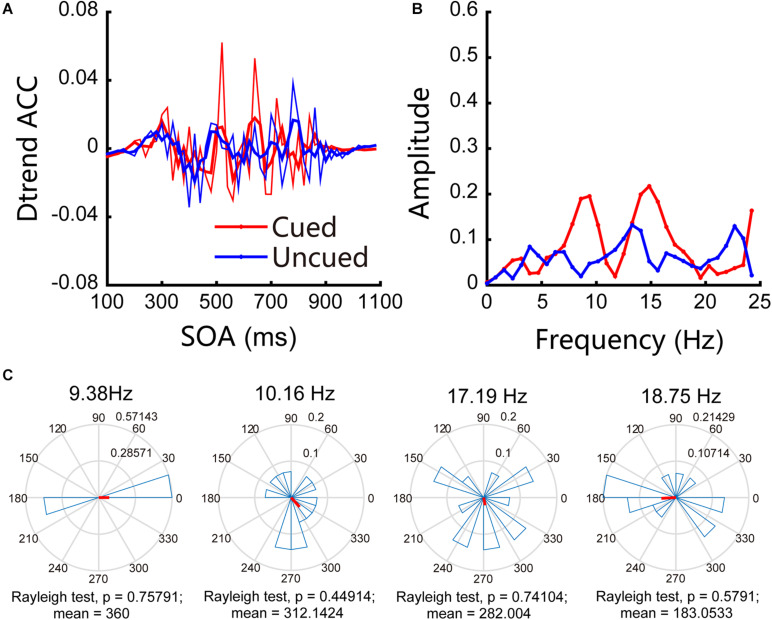
Behavioral oscillations under near cortical distance conditions. **(A)** Under both cued and uncued conditions, accuracy changed with SOA. **(B)** Amplitude of each frequency band under cued and uncued conditions. **(C)** Phase coherence between the cued and uncued conditions. Each subject’s phase difference is plotted on the circle; the average difference is plotted.

In Experiment 2, we examined attentional rhythms under near cortical distance conditions. The accuracies obtained for cued and uncued conditions were 58.55% and 56.02%, respectively, and the paired *t*-test result was *t*(13) = 2.55, *p* = 0.024 < 0.05, *d* = 0.21. The accuracies obtained for the cued and uncued locations showed that there were no significant frequency bands in the experiment. These results are shown in Figure 4A, in which the red line indicates the changes in accuracy for the cued locations over time, the blue line represents the changes in accuracy for the uncued locations over time, and the thin line represents the original accuracy; the thick line was generated through three-point smoothing. The Fourier analysis and permutation test results indicate that no significant oscillations were observed under near cortical distance conditions (see Figure 4B for details). Figure 4C shows the results of phase analysis. The phase relationships were not significant.

## Discussion

In this study, we modulated the cortical distances of two gratings while fixing the corresponding retinal distance by setting the gratings on different sides (cortically far, Experiment 1) or on the same side (cortically near, Experiment 2) of the vertical median, to investigate the interhemispheric divide effect in attentional rhythms. Our goal was to investigate the roles played by visual cortical areas during attentional rhythms. In both experiments, the retinal distance between the two critical gratings was 5.6°; however, in Experiment 1, the two gratings were presented to neurons located contralaterally on opposite sides of the VM and that therefore projected to the left and right sides of the visual cortex (a far cortical distance), whereas in Experiment 2, the two gratings were located on the right of VM and were presented to neurons that projected to the visual cortical areas on the same side (a near cortical distance). The results showed the following: (1) under contralateral presentation conditions (Experiment 1), attentional rhythms were observed, and the primary frequencies were the theta and alpha frequency bands, while (2) under near cortical distance conditions (Experiment 2), attentional rhythms were not observed. These results indicate that attentional rhythms are modulated by visual areas with an interhemispheric divide effect; hence, the periodicity of attention may be modulated by the location of the gratings relative to the physical world.

The results of Experiment 1 showed that under far cortical distance conditions, attentional rhythms manifested as low-frequency bands, consistent with the results obtained by Landau and Fries (2012), Fiebelkorn et al. (2013) and Song et al. (2014) using the cue-target paradigm (Landau and Fries, 2012; Fiebelkorn et al., 2013; Song et al., 2014). In these studies, as well as in Experiment 1 of the present study, stimuli were presented on opposite sides of the VM, and the results indicated that presentation of the stimuli at these locations enabled the stable observation of attentional rhythms in the behavioral data. However, when the retinal distance of the stimulus remained constant and the projected cortical distance was shortened, the oscillation pattern disappeared. Dugué et al. (2016) used transcranial magnetic stimulation (TMS) to interfere with the V1/V2 visual cortical regions and found that these two visual regions are involved in attentional discreteness (Dugué et al., 2016). Carlson et al. (2007) also found that visual regions play critical roles in selective attentional tracking (Carlson et al., 2007). The results in the present study suggest the important roles played by the visual cortical areas which correlate with the interhemispheric divide effect. It is worth noting that under near cortical distance conditions (Experiment 2), no attentional rhythms were observed. There are three possible reasons for this finding: first, when the cortical distance is short enough, the attention system may process the two locations as if they fall into the same attention window rather than alternating between the two locations, similar to the results reported for attentional discreteness during visual search tasks (Dugué et al., 2015); second, under shorter projected cortical distance conditions, it is difficult for attention to regularly switch between the two locations; and third, attention may switch back and forth between the two objects under these conditions, but if the cortical distance is too short, the attention switching may be too rapid to observe. Regardless of which underlying mechanism explains the obtained results of Experiment 2, these results demonstrate that cortical distance modulates attentional rhythms.

Consistent with the results reported in previous studies, attentional rhythms were observed at theta and alpha frequencies in this study. Fluctuations in RTs were correlated with theta frequencies in the visual cortex of macaques and in the frontoparietal network of pharmacoresistant epilepsy patients during distributed attention (Helfrich et al., 2018; Kienitz et al., 2018; Spyropoulos et al., 2018). The theta phase coordinated the functional interactions between the lateral intraparietal area and the frontal eye fields, and this could account for the macaque’s rhythmic attention (Fiebelkorn et al., 2018). Alpha oscillations in electroencephalographic recordings suggested that a rhythmic sampling mechanism is active during sustained attention (Jia et al., 2017, 2019). In Experiment 1, theta and alpha rhythms were also found during attention. The evidence above suggests that theta and alpha neural oscillations could be the mechanisms underlying the attentional rhythms.

The results of this study not only support the “blinking spotlight” theory of attention but also suggest that cortical distance but not physical distance modulates attentional rhythms. It is worth noting that to avoid gratings 3 and 4 attracting overt attention and creating unwanted, involuntary eye movement, we presented two additional stimuli, grating 1 and grating 2, in opposite quadrants. Although we consider this experimental design to be “optimized,” the current results may have been affected by the presentation of these two additional stimuli. The limitation of the study is that we did not monitor and measure eye movements by employing eye tracking techniques. It is important to note that the current findings are valid only if the subjects did not move their eyes. Future research should investigate attentional rhythms in situations in which only two stimuli (gratings 3 and 4) are presented while participants’ eye movements are monitored. Furthermore, considering that attentional rhythms are more evident in the right visual field than in the left visual field (Landau and Fries, 2012), we measured attentional rhythms only in the right visual field, not in the left visual field, when the cortical distance was near. In general, we found that there were no attentional rhythms when the two stimuli were in the right field of view and the cortical distance was near; however, the situation in the left visual field also requires investigation in the future. Attentional rhythms in the left visual field could differ from those in the right visual field. Previous studies have found that task difficulty can modulate attentional rhythms (Chen et al., 2017b) and that there may be different attentional rhythms regarding the same object and different objects (Fiebelkorn et al., 2013) in the visual modality. Moreover, in the auditory modality, different frequencies were involved in auditory sensitivity and decision criteria (Ho et al., 2017), and auditory perceptual history was shown to modulate alpha rhythm (Ho et al., 2019). Therefore, multiple mechanisms may underlie attentional rhythms, or the attentional rhythms could be quite flexible.

Finally, we speculate that the interhemispheric divide effect may provide some hints about the neural site of attentional rhythms. Contralateral projection patterns are consistently maintained across lower visual cortical areas (likely V1, V2, and V3) but give way to bilateral processing at higher levels in the inferotemporal and parietal cortex due to the larger visual receptive field and higher efficiency of corpus callosum transfer (Pillow and Rubin, 2002; Bullier, 2004; Alvarez and Cavanagh, 2005). Researchers have utilized the interhemispheric divide effect to investigate the role played by visual cortical areas in multiple visual phenomena, including visual crowding, illusion contours, attentive tracking, and attentional selection (Pillow and Rubin, 2002; Alvarez and Cavanagh, 2005; Carlson et al., 2007; Liu et al., 2009). The difference in the results obtained in Experiment 1 and Experiment 2 therefore suggests that the neural site of attentional rhythms may involve visual cortical areas (likely V1, V2, and V3). It should be pointed out that because we did not perform neuroimaging (fMRI or single cell recording), it is impossible to fully determine that the interhemispheric effect is due to early cortical areas such as V1, V2, and V3. Thus, we can only speculate that the interhemispheric effect may be attributed to early cortical areas such as V1, V2, and V3. Whether the role of early visual regions (likely V1, V2, and V3) will remain robust in other situations is unknown. In future studies, the role played by early visual regions should be further characterized in different situations (e.g., left visual field, object-based attention, auditory modality, and others).

## Data Availability Statement

The original contributions presented in the study are included in the article/supplementary material, further inquiries can be directed to the corresponding authors.

## Ethics Statement

The studies involving human participants were reviewed and approved by Soochow University. The patients/participants provided their written informed consent to participate in this study.

## Author Contributions

AC and MZ had the idea for the article. AC performed the research. AC and BD contributed to data analysis and to the original manuscript. All authors contributed to the drafting and revision of the manuscript.

## Conflict of Interest

The authors declare that the research was conducted in the absence of any commercial or financial relationships that could be construed as a potential conflict of interest.
